# Cytoprotective Preconditioning of Osteoblast-Like Cells with *N*-Acetyl-*L*-Cysteine for Bone Regeneration in Cell Therapy

**DOI:** 10.3390/ijms20205199

**Published:** 2019-10-20

**Authors:** Masahiro Yamada, Jun Watanabe, Takeshi Ueno, Takahiro Ogawa, Hiroshi Egusa

**Affiliations:** 1 Division of Molecular and Regenerative Prosthodontics, Tohoku University Graduate School of Dentistry, Sendai, Miyagi 980-8575, Japan; jun.watanabe.b4@tohoku.ac.jp (J.W.); egu@tohoku.ac.jp (H.E.); 2 The Jane and Jerry Weintraub Center for Reconstructive Biotechnology, Division of Advanced Prosthodontics, UCLA School of Dentistry, Los Angeles, CA 90095-1668, USA; takepro1@tmd.ac.jp (T.U.); togawa@dentistry.ucla.edu (T.O.); 3 Removable Partial Prosthodontics, Department of Masticatory Function Rehabilitation, Tokyo Medical and Dental University, Bunkyo-ku, Tokyo 113-8510, Japan; 4 Center for Advanced Stem Cell and Regenerative Research, Tohoku University Graduate School of Dentistry, Sendai, Miyagi 980-8575, Japan

**Keywords:** acute inflammation, apoptosis, cell fate, glutathione, oxidative stress

## Abstract

Oxidative stress hinders tissue regeneration in cell therapy by inducing apoptosis and dysfunction in transplanted cells. *N*-acetyl-*L*-cysteine (NAC) reinforces cellular antioxidant capabilities by increasing a major cellular endogenous antioxidant molecule, glutathione, and promotes osteogenic differentiation. This study investigates the effects of pretreatment of osteoblast-like cells with NAC on oxidative stress-induced apoptosis and dysfunction and bone regeneration in local transplants. Rat femur bone marrow-derived osteoblast-like cells preincubated for 3 h with and without 5 mM NAC were cultured in a NAC-free osteogenic differentiation medium with continuous exposure to 50 μM hydrogen peroxide to induce oxidative stress. NAC preincubation prevented disruption of intracellular redox balance and alleviated apoptosis and negative impact on osteogenic differentiation, even under oxidative stress. Autologous osteoblast-like cells with and without NAC pretreatment in a collagen sponge vehicle were implanted in critical-size defects in rat femurs. In the third week, NAC-pretreated cells yielded complete defect closure with significantly matured lamellar bone tissue in contrast with poor bone healing by cells without pretreatment. Cell-tracking analysis demonstrated direct bone deposition by transplanted cells pretreated with NAC. Pretreatment of osteoblast-like cells with NAC enhances bone regeneration in local transplantation by preventing oxidative stress-induced apoptosis and dysfunction at the transplanted site.

## 1. Introduction

Local transplantation of mesenchymal stem cells (MSCs) is one of the effective therapies for bone regeneration in orthopedics and dentistry [1,2]. MSCs have pleiotropic actions including recruitment of other MSCs and progenitor cells, angiogenesis, immunoregulatory effects, anti-apoptotic effects, and promotion of host-cell differentiation into tissue-forming cells [3]. Transplantation of naive MSC is therapeutically purposed to modulate the regenerative microenvironment [4,5,6], whereas implantation of cultured osteoblasts derived from MSCs is also explored to supply bone-forming cells with osteoinductive properties [7,8,9,10].

The effectiveness of cell transplantation on tissue regeneration still has room for improvement [11,12]. One of the critical issues is loss of transplanted cell viability after local transplantation [1,13,14]. Regardless of the types of donor cells or recipient tissue, the number of transplanted cells is reduced by more than 50% in 3 days after transplantation, and the cells barely maintain trackability for 2–3 weeks [1,14,15]. The reduced number of transplanted cells compromises the effectiveness of tissue regeneration [16,17]. In addition, loss of viability of transplanted cells fails to track the fate of the transplanted cells in local tissue. Locally transplanted osteoblast-like cells in a femoral bone defect were found to remain at the transplantation site without migrating to other organs, but showed no evidence to participate in bone regeneration [18]. Improvement of viability of transplanted cells would lead to not only enhancing the effectiveness of tissue regeneration but also clarifying the cell fate after local transplantation.

Physical tissue damage in association with transplantation procedures activates innate immune cells such as neutrophils and evokes acute inflammation by producing various proinflammatory mediators, cytokines, and reactive oxygen species (ROS) [19]. Excessive ROS generation causes oxidative stress on transplanted cells by disturbing the intracellular redox balance, eventually inducing apoptosis [5]. Oxidative stress resulting from noninfectious acute inflammation can cause the elimination of transplanted stem cells [6,20]. In addition, oxidative stress hinders the osteogenic differentiation of MSC and osteoblast progenitors [21]. The lineage commitment of MSCs is regulated toward adipocytes rather than osteoblasts under oxidative stress [22]. Addition of exogenous hydrogen peroxide (H_2_O_2_) suppressed the osteogenic differentiation of cultured human bone marrow-derived MSCs (BMSCs) [23] and murine osteoblast progenitors [24]. Regulation of cellular redox balance is one of the key factors to prevent loss of viability and dysfunctions of transplanted cells after local transplantation.

*N*-acetyl-*L*-cysteine (NAC) is an amino acid-derivative with intra- and extracellular antioxidant capabilities [25]. The functional moiety, the sulfhydryl group, of NAC can directly scavenge extracellular ROS or certain cytotoxic substances. In addition, NAC undergoes cellular uptake through solute carrier transporters [26,27] and deacetylation within the cytoplasm into a precursor, *L*-cysteine, of a major intracellular antioxidant molecule, glutathione (GSH) [28]. Exogenous GSH or *L*-cysteine is unabsorbable or unstable outside the cell, respectively. NAC is a representative antioxidant molecule to supply intracellular GSH. NAC has multiple pharmacological effects on osteoblast lineage cells in association with antioxidant capabilities [24]. NAC is proven to assist bone regeneration on an implant biomaterial by preventing wound infection [29] and improving the biomaterial cytocompatibility [30,31]. Moreover, NAC demonstrated the ability to function as an osteogenic-enhancing molecule, not to induce the differentiation of BMSCs into osteoblast progenitor cells but to promote the osteogenic differentiation of osteoblast-like cells [32]. Promotion of osteogenic differentiation by NAC may be associated with an increase of cellular GSH through uptake of NAC [33]. Moreover, preconditioning of naive BMSCs with NAC has been demonstrated to enhance bone regeneration after the local transplantation of autologous BMSCs to a massive femur defect [34]. The underlying mechanism was to keep high cellular GSH levels in BMSCs long enough to prevent apoptosis and senescence under oxidative condition during acute inflammation phase. Notably, the surviving BMSCs after local transplantation into a bone defect were found near the newly formed bone tissue but not within osteocyte lacunae [34]. This observation suggests that preconditioning with NAC protected transplanted BMSCs from oxidative stress after local transplantation and that the locally transplanted naive BMSCs were involved in bone regeneration through modulation of the regenerative microenvironment rather than differentiation into osteoblasts.

NAC’s pharmacological effects on BMSCs and osteoblast-like cells raise intriguing questions about local cell transplantation for bone regeneration. The first question is whether preconditioning with NAC is effective for bone regeneration with local transplantation of osteoblasts. The second one is whether the transplanted osteoblasts are involved in bone regeneration as bone-forming cells. NAC improves viability and differentiation of osteoblast-like cells on various bone biomaterials such as the polymethyl-methacrylate resin [30,35] and calcium phosphate-based bone substitutes [31,36], and collagen matrix scaffolds [37,38] by alleviating oxidative stress. It has been hypothesized that preconditioning osteoblasts with NAC prevents suppression of cell viability and differentiation under oxidative stress and promotes bone regeneration after local transplantation in a bone defect by activating bone-forming abilities of the transplanted osteoblasts. The purposes of this study were to examine the effects of incubating osteoblast-like cells with NAC on apoptosis and suppression of osteogenic differentiation induced by oxidative stress and to determine whether preconditioning osteoblast-like cells with NAC enhances bone regeneration in local cell transplantation in a critical-size bone defect. The histological fate of transplanted osteoblast-like cells is also discussed.

## 2. Results

### 2.1. Determination of the Concentration and Incubation Time of NAC Preconditioning for Osteoblast-Like Cells

**Figure 1:**
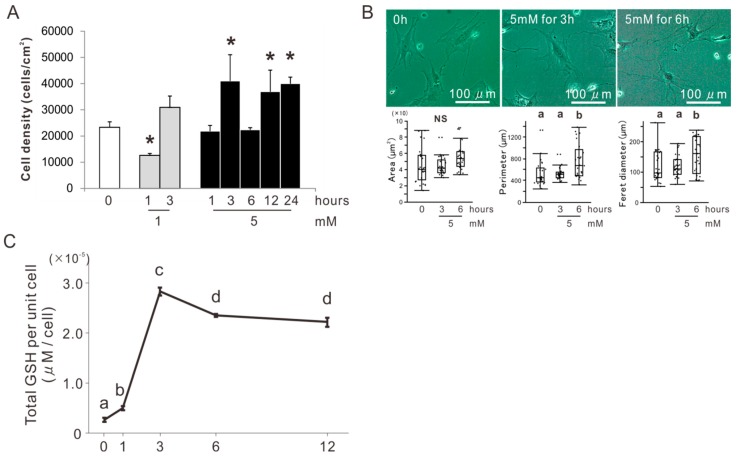
Determination of the concentration and incubation time of *N*-acetyl-*L*-cysteine (NAC) preconditioning for osteoblast-like cells. (**A**) Attached cell density on day 1 culture of rat femur bone marrow-derived osteoblast-like cells preincubated with and without 1 or 5 mM NAC up to 24 h. (**B**) Phase microscopic images and cytomorphometry for area, perimeter, and Feret diameter on day 1 culture of cells preincubated with and without 5 mM NAC up to 6 h. (**C**) Total glutathione (GSH) per unit cell in cell suspension with co-incubation with 5 mM NAC for 0, 1, 3, 6, and 12 h. The data are expressed as the mean ± standard deviation in 1A and C (*n* = 3) or the box plots overlaid with dot plots in 1B (*n* = 25). Asterisks in 1A indicate statistically significant differences between cultures co-incubated with 0 mM NAC versus 1 mM NAC for 1 h and 5 mM NAC for 3, 12, and 24 h (*p* < 0.05, Dunnett’s test). Different characters in 1B and C indicate statistically significant differences between them (*p* < 0.05, Tukey’s HSD test). NS in 1B indicates no statistically significant differences between cultures co-incubated with 5 mM NAC for 0, 3, and 6 h (*p* > 0.05, Tukey’s HSD test).

BMSCs were isolated from the femur of an 8-week-old Sprague Dawley (SD) rat (Nippon SLC, Shizuoka, Japan) and cultured for osteogenic induction in an osteogenic differentiation medium (ODM) for 2 weeks. The osteoblast-like cells were co-incubated with 0, 1, or 5 mM NAC for 1, 3, 6, 12, or 24 h in the 24 h preincubation and then cultured in a minimum essential growth medium (GM) on a culture-grade polystyrene plate. The cell density at day 1 was the highest in the culture preincubated with 5 mM NAC for 3, 12, or 24 h twice that of the culture without NAC preincubation (Dunnett’s test, *p* < 0.05) (Figure 1A). Phase contrast microscopic observation on the day 1 cultures showed polygonal cells in the culture preincubated with 5 mM NAC for 0, 3, or 6 h (Figure 1B). The cells preincubated with 5 mM NAC for 6 h tended to develop cellular projections more than the cells preincubated with 5 mM NAC for 3 h or the cells without NAC preincubation. Cytomorphometry demonstrated that there was no significant differences in area, but that perimeter and Feret diameter was higher in the cells preincubated with 5 mM NAC for 6 h than the cells preincubated with 5 mM NAC for 3 h or the cells without NAC preincubation (Tukey’s honest significant difference (HSD) test, *p* < 0.05). A GSH assay targeted at the cell suspensions co-incubated with 5 mM NAC for 0, 3, 6, or 12 h in the 12 h preincubation showed that the total GSH per unit cell reached its maximum value in 3 h and then dropped slightly (Tukey’s HSD test, *p* < 0.05) (Figure 1C).

### 2.2. Effects of Preconditioning Osteoblast-Like Cells with NAC on Cell Viability under Oxidative Stress

**Figure 2:**
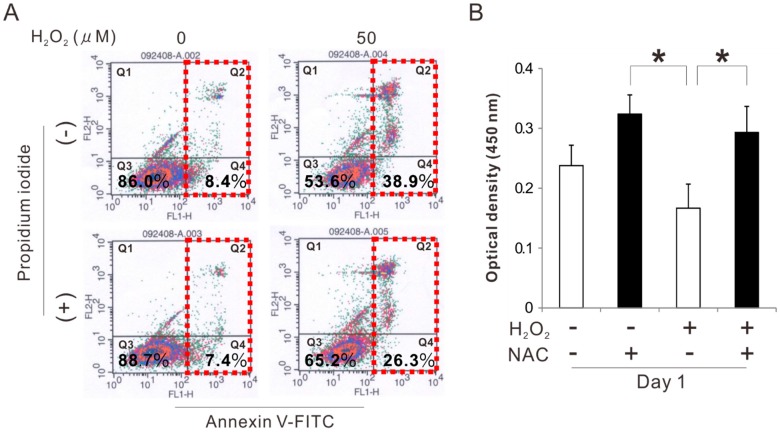
Effects of preconditioning osteoblast-like cells with *N*-acetyl-*L*-cysteine (NAC) on cell viability under oxidative stress. Rat femur bone marrow-derived osteoblast-like cells were preincubated with or without 5 mM NAC for 3 h and then cultured in an osteogenic differentiation medium with and without exposure to 50 μM H_2_O_2_. (**A**) Flow cytometry analysis for annexin V-fluorescein isothiocynate (FITC) and propidium iodide in the culture 24 h after seeding. The percentage of viable cells (Q3: quadrant 3); necrotic cells (Q1: quadrant 1); and apoptotic cells (within red-dashed enclosure), which is the sum of early (Q4: quadrant 4) and late (Q2: quadrant 2) apoptotic cells, is shown within each quadrant of the flow cytometric images. (**B**) Attached cell number evaluated by WST-1 colorimetry assay on day 1 culture. The data represent the mean ± standard deviation (*n* = 3). Asterisks in 2B indicate statistically significant differences (*p* < 0.05, Tukey’s HSD test).

Rat femur bone marrow-derived osteoblast-like cells preincubated with 5 mM NAC for 3 h were cultured on polystyrene culture plates in an ODM with and without 50 μM H_2_O_2_ as an oxidative stress inducer. Flow cytometry analysis with annexin V-fluorescein isothiocynate (FITC) and propidium iodide staining 24 h after seeding showed that the percentage of viable cells in the cell population was reduced from 86% to 54% because of exposure to H_2_O_2_ (Figure 2A). In addition, exposure to H_2_O_2_ increased the percentage of apoptotic cells from 8% to approximately 40%. By contrast, the culture preincubated with NAC reduced apoptosis induced by exposure to H_2_O_2_, with the percentage of viable and apoptotic cells reaching 65% and 26%, respectively. Preincubation with NAC by itself did not affect apoptotic induction. The number of attached cells on day 1 was decreased because of exposure to H_2_O_2_. By contrast, preincubation with NAC increased the value regardless of the exposure to H_2_O_2_ (Tukey’s HSD test, *p* < 0.05) (Figure 2B).

### 2.3. Effects of Preconditioning Osteoblast-Like Cells with NAC on Cellular Redox Balance under Oxidative Stress

**Figure 3:**
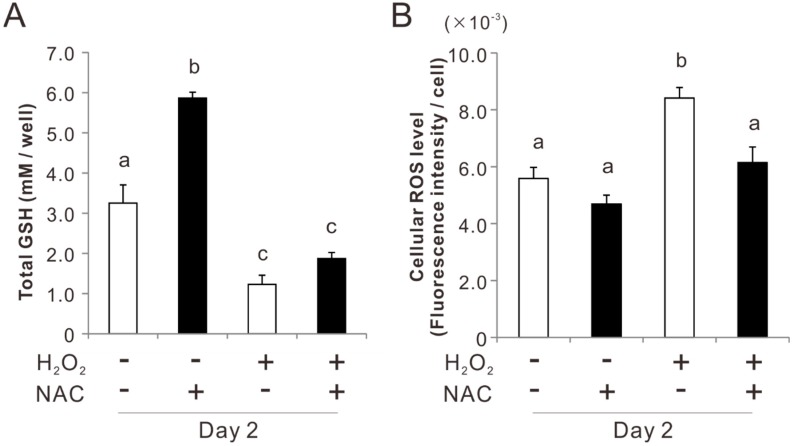
Effects of preconditioning osteoblast-like cells with *N*-acetyl-*L*-cysteine (NAC) on cellular redox balance under oxidative stress. Rat femur bone marrow-derived osteoblast-like cells were preincubated with or without 5 mM NAC for 3 h and then cultured in an osteogenic differentiation medium with and without exposure to 50 μM H_2_O_2_ for 2 days. Cellular total glutathione (GSH) levels (**A**) and intracellular reactive oxygen species (ROS) levels (**B**) in each condition were measured by a fluorometric or photometric analysis. The data represent the mean ± standard deviation (*n* = 3). Different characters indicate statistically significant differences between them (*p* < 0.05, Tukey’s HSD test).

Preincubation with 5 mM NAC for 3 h doubled the total GSH in rat femur bone marrow-derived osteoblast-like cells on day 2 (Tukey’s HSD test, *p* < 0.05) (Figure 3A). After exposure to 50 μM H_2_O_2_ for 2 days, the GSH level was reduced by 70% in both the cultures with and without NAC preincubation. Detection of cellular ROS with a membrane-permeable and ROS-reactive fluorescent agent (2′,7′-dichlorodihydrofluorescein diacetate) showed that the cellular ROS level in the culture after exposure to 50 μM H_2_O_2_ for 2 days increased 1.5 times (Tukey’s HSD test, *p* < 0.05) (Figure 3B). By contrast, the culture preincubated with NAC did not change the cellular ROS level regardless of exposure to H_2_O_2_ (*p* > 0.05).

### 2.4. Effects of Preconditioning Osteoblast-Like Cells with NAC on Proliferation under Oxidative Stress

**Figure 4:**
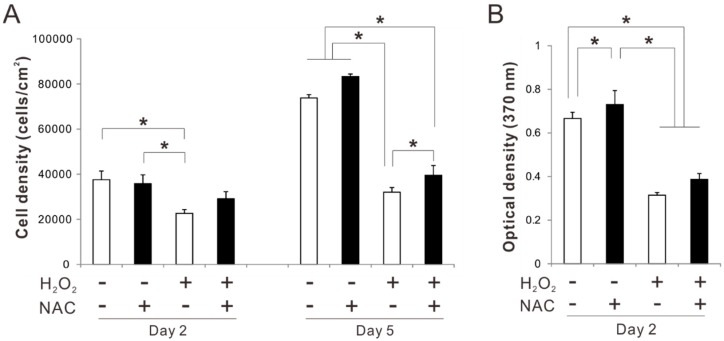
Effects of preconditioning osteoblast-like cells with *N*-acetyl-*L*-cysteine (NAC) on proliferation under oxidative stress. Rat femur bone marrow-derived osteoblast-like cells were preincubated with or without 5 mM NAC for 3 h and then cultured in an osteogenic differentiation medium with and without exposure to 50 μM H_2_O_2_ for 2 or 5 days. Attached cell numbers (**A**) and proliferative activity (**B**) in each condition were measured by cell-counting with a hematocytometer and bromodeoxyuridine, 5-bromo-2′-deoxyuridine-incorporation assay, respectively. The data represent the mean ± standard deviation (*n* = 3). Asterisks indicate statistically significant differences (*p* < 0.05, Tukey’s HSD test).

Exposure to H_2_O_2_ for 2 days significantly reduced the cell density of the rat femur bone marrow-derived osteoblastic cell culture without NAC preincubation (Tukey’s HSD test, *p* < 0.05) (Figure 4A), whereas, the values did not decrease in the culture preincubated with NAC regardless of the exposure to H_2_O_2_ (*p* > 0.05). Similarly, cell density in the culture preincubated with NAC was higher on day 5 than in the culture without NAC preincubation (Tukey’s HSD test, *p* < 0.05) (Figure 4A), even after a significant reduction because of exposure to H_2_O_2_. A 5-bromo-2’-deoxyuridine (BrdU) incorporation assay for cell proliferation showed that proliferative activity in the culture preincubated with NAC was higher on day 2 than in the culture without NAC preincubation (Tukey’s HSD test, *p* < 0.05) (Figure 4B). Proliferative activity was reduced by exposure to H_2_O_2_ regardless of NAC preincubation.

### 2.5. Effects of Preconditioning Osteoblast-Like Cells with NAC on Osteogenic Differentiation under Oxidative Stress

**Figure 5:**
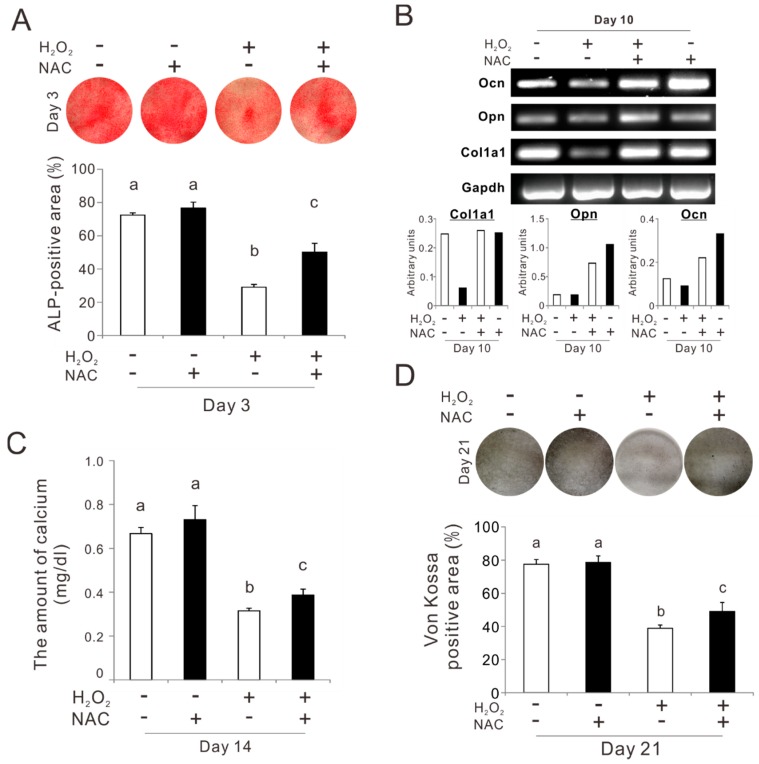
Effects of preconditioning osteoblast-like cells with *N*-acetyl-*L*-cysteine (NAC) on osteogenic differentiation under oxidative stress. Rat femur bone marrow-derived osteoblast-like cells were preincubated with or without 5 mM NAC for 3 h and then cultured in an osteogenic differentiation medium with and without exposure to 50 μM H_2_O_2_. (**A**) Alkaline phosphatase (ALP) activity was evaluated on day 3 culture with an azo-dye staining. The upper and lower panels in 5A are the representative images of stained cultures and the histogram of the percentage of the ALP-positive area relative to total cell growth area on a culture well, respectively. (**B**) Gene expressions of bone tissue-related extracellular matrix such as pro-alpha1 chains of type I collagen (*col1a1*), osteopontin (*opn*), and osteocalcin (*ocn*) with glyceraldehyde-3-phosphate dehydrogenase (*gapdh*) as a house-keeping gene were evaluated on day 10 culture with reverse transcription polymerase chain reaction (RT-PCR). Upper and lower panels in (**B**) are the representative PCR bands visualized with ethidium bromide staining and the histogram of the expression levels of each gene standardized by gapdh expression. Matrix mineralization was evaluated on days 14 and 21 with a titration calorimetry for calcium ions extracted from the cultured extracellular matrix (**C**) and Von Kossa staining (**D**), respectively. Upper and lower panels in (**D**) are the representative images of stained cultures and the histogram of the percentage of the Von Kossa-positive area relative to total cell growth area on a culture well, respectively. The data represent the mean ± standard deviation (*n* = 3). Different characters indicate statistically significant differences between them (*p* < 0.05, Tukey’s HSD test).

Rat femur bone marrow-derived osteoblast-like cells preincubated with 5 mM NAC for 3 h were cultured for up to 21 days in an ODM on a culture-grade polystyrene plate under consistent exposure to 50 μM H_2_O_2_. The percentage of the alkaline phosphatase (ALP)-positive area on day 3 was reduced because of exposure to 50 μM H_2_O_2_ from 70% to 30% (Tukey’s HSD test, *p* < 0.05) (Figure 5A). Preincubation with NAC improved the value in the culture after exposure to H_2_O_2_, to 50% (Tukey’s HSD test, *p* < 0.05). A reverse transcriptase polymerase chain reaction (RT-PCR) analysis on day 10 showed that exposure to H_2_O_2_ downregulated the expression of the bone-related markers such as pro-alpha1 chains of type I collagen (*col1a1*) and osteocalcin (*ocn*) in the culture (Figure 5B). Preincubation with NAC prevented the reduction of *col1a1* expressions after exposure to H_2_O_2_. The expressions of osteopontin (*opn*) and ocn were upregulated by preincubation with NAC regardless of exposure to H_2_O_2_. The amount of calcium in the culture on day 14 decreased by 80% because of the exposure to H_2_O_2_ (Tukey’s HSD test, *p* < 0.05) (Figure 5C). Preincubation with NAC restored the amount of calcium in the culture after exposure to H_2_O_2,_ up to 60% of that in the cultures without exposure to H_2_O_2_ (Tukey’s HSD test, *p* < 0.05). The percentage of the Von Kossa-positive area on day 21 was reduced from 80% to 40% because of exposure to H_2_O_2_ (Tukey’s HSD test, *p* < 0.05) (Figure 5D). Preincubation with NAC significantly restored the percentage under exposure to H_2_O_2_ (Tukey’s HSD test, *p* < 0.05).

### 2.6. Effects of Preconditioning Osteoblast-Like Cells with NAC on Osteogenic Differentiation

**Figure 6:**
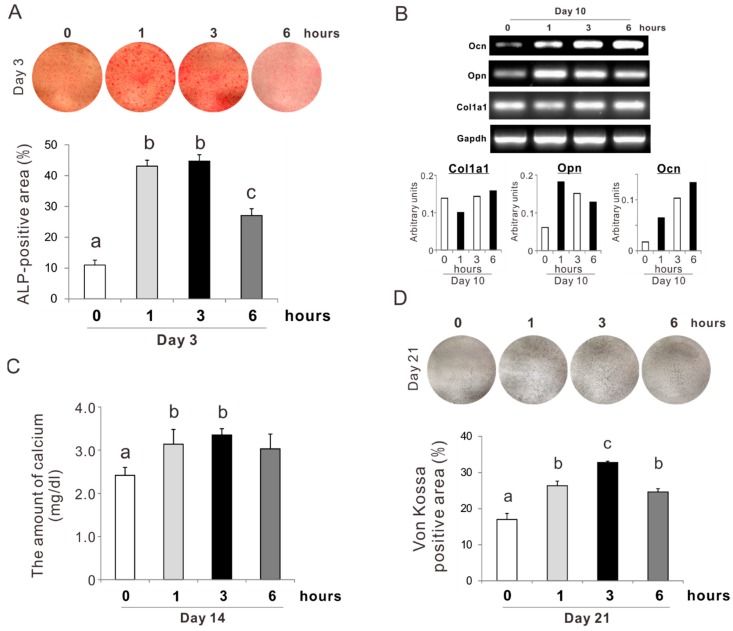
Effects of preconditioning osteoblast-like cells with *N*-acetyl-*L*-cysteine (NAC) on osteogenic differentiation. Rat femur bone marrow-derived osteoblast-like cells were preincubated with or without 5 mM NAC for 1, 3, and 6 h and then cultured in an osteogenic differentiation medium without dexamethasone. (**A**) Alkaline phosphatase (ALP) activity was evaluated on day 3 culture with an azo-dye staining. The upper and lower panels in (A) are the representative images of stained cultures and the histogram of the percentage of ALP-positive area relative to total cell growth area on a culture well, respectively. (**B**) Gene expressions of bone tissue-related extracellular matrix such as pro-alpha1 chains of type I collagen (*col1a1*), osteopontin (*opn*), and osteocalcin (*ocn*) with glyceraldehyde-3-phosphate dehydrogenase (*gapdh*) as a house-keeping gene were evaluated on day 10 of culture with reverse transcription polymerase chain reaction. Upper and lower panels in (B) are the representative PCR bands visualized with ethidium bromide staining and the histogram of the expression levels of each gene standardized by gapdh expression. Matrix mineralization was evaluated on days 14 and 21 with a titration calorimetry for calcium ions extracted from the cultured extracellular matrix (**C**) and Von Kossa staining (**D**), respectively. The upper and lower panels in (D) are the representative images of stained cultures and the histogram of the percentage of the Von Kossa-positive area relative to total cell growth area on a culture well, respectively. The data represent the mean ± standard deviation (*n* = 3). Different characters indicate statistically significant differences between them (*p* < 0.05, Tukey’s HSD test).

Rat femur bone marrow-derived osteoblast-like cells preincubated with 5 mM NAC for 0, 1, 3, and 6 h were cultured in ODM without dexamethasone on a culture-grade polystyrene plate. On day 3, the percentage of the positive area for ALP staining was the highest in the culture preincubated with NAC for 1 or 3 h (Tukey’s HSD test, *p* < 0.05) (Figure 6A). The value in the culture preincubated with NAC for 6 h was approximately half that of the culture preincubated for 1 or 3 h. Gene expressions of *opn* and *ocn* in the osteoblastic culture on day 10 appeared to be upregulated by NAC preincubation, whereas *col1a1* did not change (Figure 6B). The amount of calcium on day 14 was increased by 30% in the culture preincubated with NAC for 1 or 3 h (Tukey’s HSD test, *p* < 0.05) (Figure 6C). The value in the culture preincubated with NAC for 6 h did not differ from that in the other preincubation conditions. The percentage of the Von Kossa-positive area in the culture on day 21 was significantly increased with NAC preincubation (Figure 6D). The value was the highest in the culture preincubated with NAC for 3 h with an increase from 18% to 32% (Tukey’s HSD test, *p* < 0.05).

### 2.7. Effects of Preconditioning Osteoblast-Like Cells with NAC on Bone Regeneration in Autologous Local Cell Transplantation

**Figure 7:**
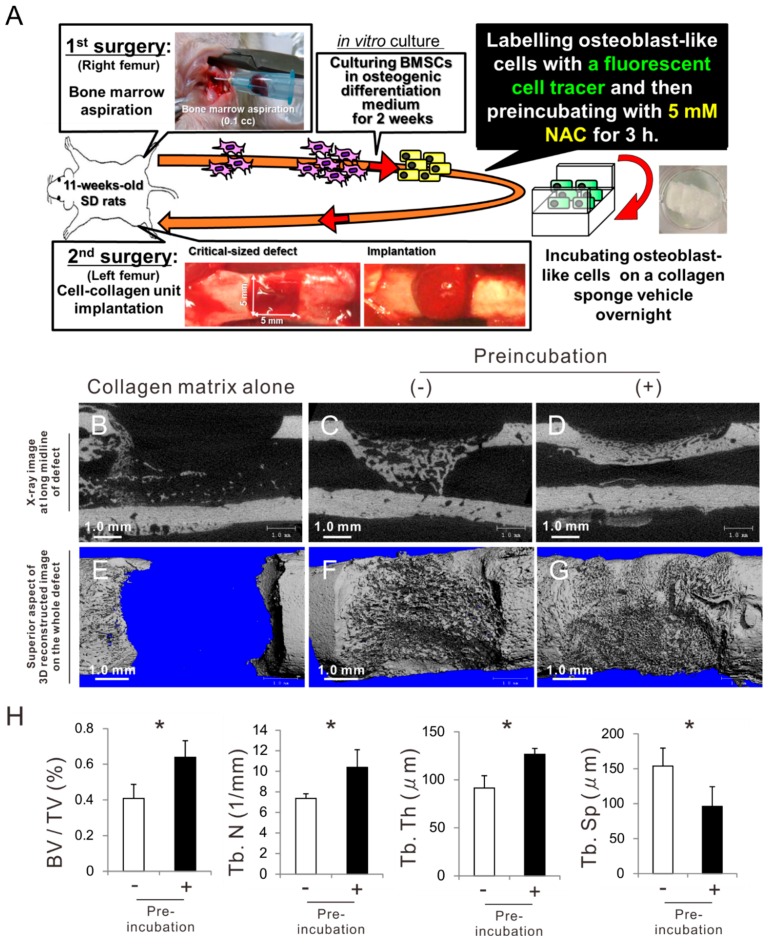
Effects of preconditioning osteoblast-like cells with *N*-acetyl-*L*-cysteine (NAC) on bone regeneration in autologous local cell transplantation. (**A**) Scheme indicating outline of the autologous local cell transplantation model. Bone marrow tissue was aspirated from the right femur bone in the first surgery. Bone marrow stromal cells (BMSCs) were cultured on a 10-cm polystyrene dish in an osteogenic differentiation medium for 2 weeks for the purpose of cell growth and differentiation into osteoblast-like cells. At confluence, the cells were labeled with a quantum-dot fluorescent tracer and then preincubated with 5 mM NAC for 3 h. The cells were seeded on a collagen sponge in a NAC-free growth medium. After overnight incubation, the cell-collagen unit was implanted into a critical-sized defect (5 × 5 mm) created on the opposite left femur bone of the donor rat. Representative micro-computed tomography images of the original grayscale cross-section at the long midline of the defect (**B**–**D**) and overhead view of constructed three-dimensional bone architecture for the defect (**E**–**G**) 3 weeks after implantation of a collagen sponge alone (**B**,**E**) or autologous rat osteoblast-like cells with (**D**,**G**) and without (**C**,**F**) preincubation with 5 mM NAC for 3 h. Quantitative assessment of three-dimensional bone morphometrical parameters (**H**), bone volume fraction (BV/TV) and trabecular number (Tb. N), thickness (Tb. Th) and separation (Tb. Sp) was performed in the areas of interest in the cortical and bone marrow space regions. The presented data represent the mean ± standard deviation (*n* = 4). Asterisks indicate statistically significant differences (*p < 0.05*, Student’s or Welch’s t-test).

BMSCs were isolated from the right femur of an 11-week-old male SD rat (Figure 7A). The cells were cultured in an ODM for 2 weeks during which they grew and differentiated into osteoblast-like cells. After the preculture, the cells were labeled with a fluorescent organic dot cell tracer for 12 h and then incubated with or without 5 mM NAC for 3 h in suspension. Subsequently, the cells underwent overnight incubation on a collagen sponge as a vehicle for cell transplantation. The cell-collagen complex or collagen sponge alone was implanted into a critical-size cortical bone defect created on the center of the left femur of the same rat. The bone defects did not allow cortical bone healing with implantation of the collagen sponge alone without cells at 3 weeks postoperatively (Figure 7B,E). The collagen sponge containing osteoblast-like cells without NAC preincubation formed limited and sparse mineralized structures in the bone marrow space beneath the defects and did not completely close the cortical bone defects (Figure 7C,F). By contrast, the cortical bone defects filled with the collagen sponge containing osteoblast-like cells preincubated with 5 mM NAC for 3 h were completely closed, with a dense and contiguous mineralized structure (Figure 7D,G). A bone morphometric analysis was intended for the area of interest defined as the region surrounded by connecting external basic lamellar points of both sides of the defect edges and the corresponding points on the opposite internal basic lamellar surface. Mineralized structures in the bone defects implanted with osteoblast-like cells preincubated with NAC were significantly superior to those in the defects implanted with cells without NAC preincubation in terms of bone morphological parameters such as bone volume fraction (BV/TV) and trabecular number (Tb. N), thickness (Tb. Th), and separation (Tb. Sp) (Figure 7H) (Student’s or Welch’s t-test, *p* < 0.05).

### 2.8. Histological Features of Newly Formed Bone Tissue after Autologous Local Transplantation of Osteoblast-Like Cells with NAC

**Figure 8:**
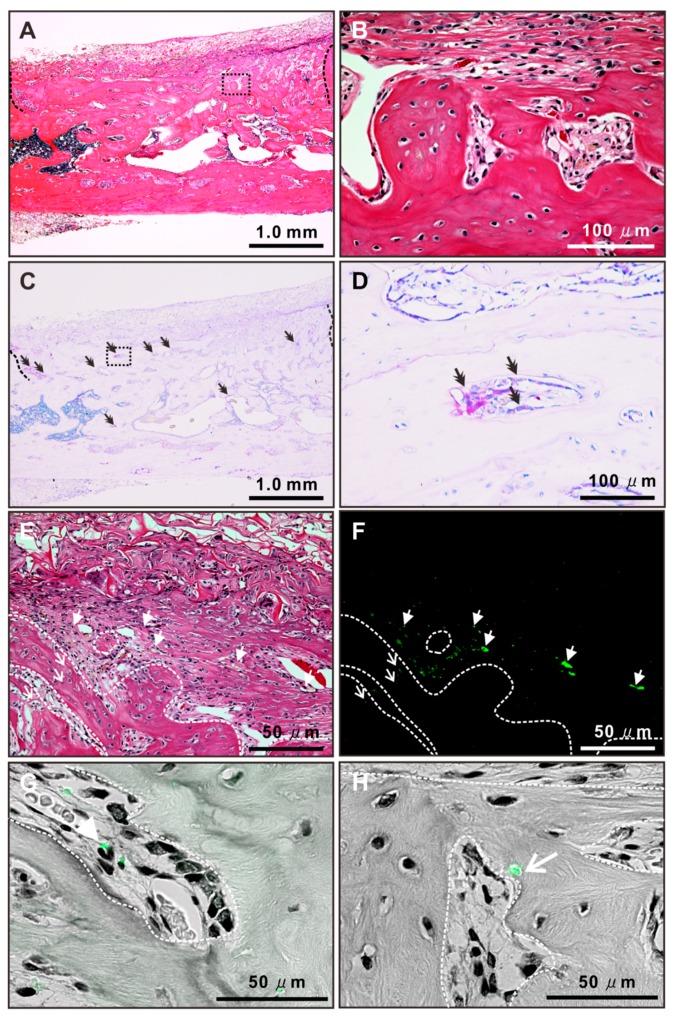
Histological features of newly formed bone tissue after autologous local transplantation of osteoblast-like cells with *N*-acetyl-*L*-cysteine (NAC). Representative light-microscope images with hematoxylin and eosin (**A**,**B,E**) or tartrate-resistant acid phosphatase (TRAP) staining (**C**,**D**) and fluorescent images merged with (**G**,**H**) and without (**F**) a light-microscope image in the same region on the histological cross section at the long midline of a 5 × 5 mm defect on the rat femur cortical bone 3 weeks after local transplantation of the collagen sponge vehicle used to load autologous rat osteoblast-like cells pretreated with 5 mM NAC for 3 h. Green in the fluorescent images (**F**–**H**) indicates transplanted osteoblast-like cells prelabeled with a fluorescent cell tracer. Notes: White arrows: location of green fluorescent signals within newly formed bone tissue; white arrowheads: location of green fluorescent signals on the outside of newly formed bone tissue; double arrow: location of TRAP positive spots; black dashed line: a border between the existing and newly formed bone tissue; white dash line: surface of newly formed bone tissue; black dash square in (**A**,**C**): the regions corresponding to higher magnification images in (**B**,**D**).

The bone defect implanted with osteoblast-like cells preincubated with NAC was completely closed, with thick, compact, and contiguous bone tissue 3 weeks after surgery (Figure 8A). The newly formed bone was mature lamellar tissue with osteocytic lacunae without remnant collagen sponge materials (Figure 8B). The newly formed bone tissue was rich in tartrate-resistant acid phosphate (TRAP) staining-positive areas (double arrows in Figure 8C), where multinuclear cells were attached to the bone surface (double arrows in Figure 8D). Fluorescent positive signals were observed both within and nearby the newly formed bone tissue (arrows and arrowheads in Figure 8E,F) in the defect implanted with osteoblast-like cells preincubated with NAC. Merged images with fluorescent and bright fields showed that the fluorescent signals within the bone tissue were located either near the host osteoblast-like cells with an active form on the bone surface (arrowheads in Figure 8G) or within the osteocyte lacunae (an arrow in Figure 8H).

## 3. Discussion

This study employed nonclonal BMSCs, which contain stem cells as a subpopulation and are generally used as MSCs for therapeutic purpose [1]. The cell population may have remained heterogeneous, but it is known that the supplementation of dexamethasone, beta-glycerophosphate, and ascorbic acid into the culture media enhances the osteoblastic differentiation of rat BMSCs [39]. As shown in Figure 6, the rat BMSCs grown in the ODM for 2 weeks expressed signatures for osteoblastic differentiation, regardless of NAC preincubation. Hence, nonclonal BMSCs with osteogenic induction were regarded as osteoblast-like cells in the present study.

Our previous study showed that 5 mM NAC enhanced the cellular antioxidant capability in the rat osteoblastic cell culture, but 10 mM NAC reduced cell attachment [32]. The attached cell density on day 1 in osteoblastic cultures peaked by preconditioning with 5 mM NAC for 3, 12, or 24 h (Figure 1A). Preconditioning with 5 mM NAC for 3 h kept polygonal cell shapes that was typically observed in cultured osteoblast-like cells [40] as observed on the cells without NAC preconditioning on day 1; whereas, preconditioning for 6 h changed the cell morphology (Figure 1B). In addition, total GSH was the highest in the cells pretreated with 5 mM NAC for 3 h and decreased with the further incubation time (Figure 1C). Therefore, 5 mM and 3 h were set as the NAC preincubation concentration and time, respectively, for osteoblastic cell cultures in the present study. NAC preincubation in such conditions alleviated induction of apoptosis and reduction of cell attachment under exposure to oxidative stress (Figure 2 and Figure 3). Osteoblast-like cells under exposure to oxidative stress were compromised in cellular redox balance, which was demonstrated by a reduction of total GSH and elevation of cellular ROS. NAC preincubation did not prevent reduction of total GSH but did elevate the cellular ROS levels under exposure to oxidative stress (Figure 3). This indicated that NAC preincubation prevented a redox imbalance in osteoblast-like cells by reinforcing cellular antioxidant capability to a level high enough to resist contiguous exposure to oxidative stress.

MSCs are known to accumulate in lung tissue after systemic infusion through intravenous injection despite the homing capability to injured tissues [41]. Likewise, systematically-injected osteoblast-like cells are not located in a bone cavity but distributed in viscera such as lung or liver [18]. Various cell delivery systems and tissue-engineering technologies based on local cell transplantation have been developed for bone regeneration [2,8]. However, oxidative stress is unavoidable for transplanted cells in any implantation procedures, impairing the viability of the transplanted cells and regenerative outcomes [6,20,21,22]. Preincubation of osteoblast-like cells with 5 mM NAC for 3 h restored osteogenic differentiation suppressed by the exposure to oxidative stress, although cellular proliferation did not improve (Figure 4 and Figure 5). Under exposure to oxidative stress, NAC preincubation not only improved reductions in the areas of ALP activity and Von Kossa-positive mineralizing nodules but also prevented downregulation of bone matrix-related gene expression, which was not affected by the numbers of attached cells. In addition, osteoblast-like cells preincubated with 5 mM NAC for 3 h had the highest expressions in all osteogenic differentiation markers, such as ALP activity, bone matrix-related genes, and matrix mineralization under growth culture conditions without oxidative stress (Figure 6). Those observations indicate that NAC preincubation with optimal conditions promoted osteogenic differentiation and enhanced antioxidant capability in osteoblast-like cells.

Our previous study indicated that continuous co-incubation with 5 mM NAC did not induce differentiation of MSCs into osteoblast progenitor cells but promoted differentiation of osteoblast-like cells [32]. The present study indicated that a single treatment of NAC preincubation influenced osteoblast characteristics for several days after NAC removal while promoting osteogenic differentiation. Although it remains unclear how NAC promotes differentiation of osteoblast-like cells, indirect involvement of NAC through GSH synthesis has been suggested [33]. Ascorbic acid, which is a representative antioxidant, is known to promote osteogenic differentiation through activation of nuclear factor erythroid 2-related factor (Nrf), a redox signaling pathway [42]. Nrf proteins are transcriptional factors that not only regulate gene expressions of antioxidant molecules and enzymes in response to cellular ROS [43] but also bind to the antioxidant response sequence of *ocn* promoters [44]. Exogenous GSH synthesis by NAC preincubation may affect redox signaling pathways as a result of regulation of cellular redox balance. Preincubation with 5 mM NAC for 1 h did not substantially increase total GSH in osteoblast-like cells but promoted osteogenic differentiation (Figure 1C and Figure 6). This suggests that the effects of NAC on osteogenic differentiation are independent of GSH synthesis. NAC has the potential to activate the extracellular signal-regulated kinase-mitogen-activated protein kinase (ERK-MAPK) [45], which also consists of redox-sensitive molecules [46]. Recently, a biochemical pathway originating from NAC for intracellular production of hydrogen sulfide [47], which is a strong reductant known to control the osteoblastic function through the ERK-MAPK pathway [48], has been reported. Influences of cellular redox status and the relative molecules on osteogenic differentiation would be of great interest for future research.

**Figure 9:**
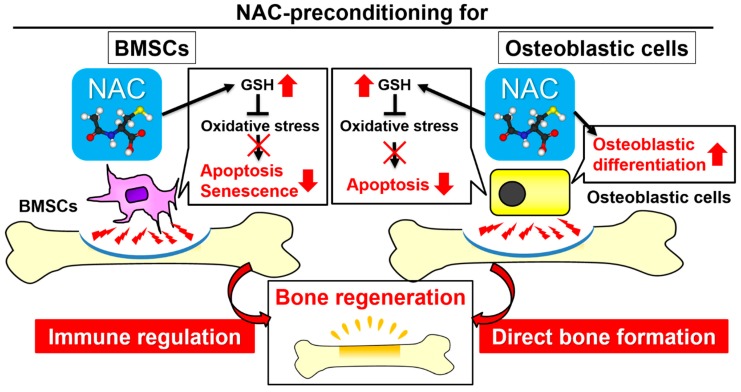
Scheme illustrating the proposed mechanism underlying the enhancement of bone regeneration by preincubation of bone marrow stromal cells (BMSCs) and osteoblast-like cells with *N*-acetyl-*L*-cysteine (NAC) for autologous local transplantation, respectively. As in BMSCs and osteoblast-like cells, uptake of NAC prevents oxidative stress-mediated apoptosis and/or cellular senescence by increasing the level of the primary antioxidant molecule, glutathione. BMSCs or osteoblast-like cells preconditioned with NAC can survive during acute inflammation in the recipient site. Surviving BMSCs are indirectly involved in bone formation through regulatory functions for the immune system, whereas surviving osteoblast-like cells are directly involved in bone deposition through enhancement of bone-forming ability with upregulated osteogenic differentiation. Pathways in BMSCs are based on our previous study [34].

In the present study, the effects of NAC as a cell preconditioning agent on bone regeneration were evaluated in an autologous local transplantation model in a critical-size defect in a rat femur. Autologous local transplantation of osteoblast-like cells formed mineralized structures in the defect to some extent, but the structures were weak and did not cover the entire cortical defect (Figure 7B). By contrast, local transplants of osteoblast-like cells preincubated with NAC yielded nearly complete defect healing in the form of a thick, compact, and contiguous mineralized structure. The mineralized structure consisted of mature lamellar bone tissue with osteoclastic activity (Figure 8A–D). Moreover, fluorescent signals meaning the transplanted cells and their daughter cells were detected within osteocytic lacunae in the newly formed bone (Figure 8E,F,H). Those observations indicate that transplanted osteoblast-like cells pretreated with NAC are involved in the formation of the normal bone tissue with homeostasis as bone-forming cells. By contrast, in the previous study using naive BMSCs in local transplants, transplanted cells were localized in the fibrous tissue adjacent to the newly formed bone tissue but not seen in osteocytic lacunae [34]. BMSCs pretreated with NAC enhanced bone regeneration as immune regulatory cells with resistance to oxidative stress-mediated apoptosis and senescence during acute inflammation [34]. NAC preconditioning markedly reinforces resistance to oxidative stress-mediated apoptosis or senescence and enhances immune regulatory function and bone-forming capability in local transplants of BMSCs and osteoblast-like cells, respectively (Figure 9).

Preconditioning BMSCs or osteoblast-like cells with NAC brought about an intriguing suggestion that the osteogenic differentiation state of MSCs before transplantation determines the fate of the transplanted cells in local tissue. However, there were some limitations in the present study. First, the terminal fate of transplanted cells was not completely elucidated because of the short observation period of 3 weeks after transplantation. In addition, the characteristics of transplanted cells may not be uniform because nonclonal BMSCs without cell sorting were used. In the present study, many surviving transplanted cells were found near the host osteoblast-like cells on the surface of the newly formed bone as well (Figure 8E–G). It remained unclear whether those surviving transplanted cells are involved in direct bone formation or modulation of the surrounded cells. Long-term follow-up of the sorted transplanted cells would clarify the role and terminal fate of transplanted cells, depending on the osteogenic differentiation state. Cell preconditioning techniques with NAC may advance transplantation technology and help clarify biological questions in stem cell medicine.

## 4. Material and Methods

All animal experiments in the present study was performed according to a protocol approved by the University of California at Los Angeles Chancellor’s Animal Research Committee and the Institutional Laboratory Animal Care and Use Committee of Tohoku University (protocol no. 2016-062, approved on 23 September 2016).

### 4.1. Reagent Preparation and Application

NAC (Sigma-Aldrich, St. Louis, MO, USA) was prepared as a 1 M working solution by dissolving in 1 M 4-(2-hydroxyethyl)-1-piperazineethanesulfonic acid (Sigma-Aldrich) buffer at pH 7.2. A final NAC concentration for cell conditioning was set as 1 or 5 mM by adding 1 or 5 μL of NAC working solution to 1 mL of cell suspension, respectively. H_2_O_2_ (Wako Pure Chemical, Osaka, Japan) was adjusted to 5 mM as a working solution with sterilized ultrapure water. The working solution of H_2_O_2_ was used for experiments immediately after preparation. A final concentration of H_2_O_2_ for cell culture was set at 50 μM by adding 10 μL of H_2_O_2_ working solution into 1 mL of culture media.

### 4.2. Osteoblastic Cell Culture

BMSCs were isolated from femurs of 8-week-old male SD rats according to the method previously described [49]. Briefly, the bone marrow tissue was flushed out from femoral cavity. The isolated BMSCs were cultured in an ODM consisting of alpha modification of eagle’s minimum essential medium (α-MEM: GIBCO^®^ Gluta MAX^TM^, no nucleosides; Thermo Fisher Scientific, MA., USA), supplemented with 10% fetal bovine serum (Gibco^TM^ FBS 10082147, Thermo Fisher Scientific), 10^−8^ M dexamethasone, 10 mM Na-beta-glycerophosphate, 50 μg/mL ascorbic acid, 100 U of penicillin, and 100 μg/mL streptomycin (Wako Pure Chemical) at 37 °C under an atmosphere of 5% CO_2_. The cell culture underwent passages two or three times in an ODM for 2 weeks. At 80% confluence, cells were harvested with 0.25% trypsin/1 mM EDTA and then underwent NAC preincubation.

The cells were suspended in a 15 mL conical polypropylene tube (TrueLine TR2001, Nippon Genetics Co.,Ltd., Tokyo, Japan) with a GM consisting of α-MEM, 10% fetal bovine serum, 100 U of penicillin, and 100 μg/mL streptomycin. The cell suspensions were processed according to each protocol of the following three experiments.

In a culture experiment to determine the concentration and treatment time of NAC for osteoblast-like cells, the suspended cells were preincubated with and without 1 or 5 mM NAC. NAC was added into the cell suspension so that co-incubation time with NAC was 1, 3, 6, 12, or 24 h in the 24 h preincubation. After preincubation, the cells were re-suspended into a NAC-free GM and seeded in 12-well culture plates at a cell density of 1.5 × 10^4^ cells/cm^2^.

In a culture experiment to determine the effect of NAC preconditioning on oxidative stress, the cells suspended with or without 5 mM NAC were preincubated for 3 h. After preincubation, the cells were re-suspended into a NAC-free ODM and seeded in 12-well culture plates at a cell density of 3.0 × 10^4^ cells/cm^2^ with and without H_2_O_2_ addition.

In a culture experiment to determine the effect of NAC preconditioning on osteoblastic differentiation of osteoblast-like cells, the suspended cells were preincubated with and without 5 mM NAC. NAC was added into the cell suspension so that co-incubation time with NAC was 1, 3, or 6 h in the 6 h preincubation. After preincubation, the cells were re-suspended into a NAC-free ODM without dexamethasone and seeded in 12-well culture plates at a cell density of 1.5 × 10^4^ cells/cm^2^.

The culture medium corresponding to each experiment was renewed at intervals of 3 days.

### 4.3. Cell Attachment, Cytomorphometry, and Proliferation Assay

The numbers of attached cells were evaluated with cell density measurements and a WST-1 assay. For cell density measurements, the attached cells were gently rinsed twice with phosphate buffered saline (PBS) on days 2 and 5 after seeding and then detached with 300 μL of 0.25% trypsin/1 mM EDTA-4Na for 15 min at 37 °C. A hematocytometer (Bright-Line, Hausser Scientific, PA, USA) was used to count the number of collected cells. The substrates were examined under a microscope to confirm no remaining cells. For the WST-1 assay, the culture medium was replaced on day 1 after seeding with a fresh medium containing a WST-1 reagent (Roche Diagnostics, Indianapolis, IN, USA). Then, the cells were incubated for 3 h at 37 °C under an atmosphere of 5% CO_2_. After gentle shaking, the optical density (OD) for formazan in the supernatant was measured at 450 nm in the microplate reader, which had a linear relationship with the number of cells attached on substrates.

Cytomorphometry was performed on phase contract microscopic images in the day 1 culture using an image analyzer (ImageJ, NIH, ML, USA). For each culture, 25 cells were randomly selected and analyzed for area, perimeter, and Feret diameter.

Cellular proliferation activity was evaluated with BrdU incorporation assay (Sigma-Aldrich). On day 2, the culture medium with H_2_O_2_ was renewed into a fresh GM containing 100 mM BrdU solution and then the cells were incubated for additional 10 h. After DNA was denatured, cultures were incubated with anti-BrdU conjugated with peroxidase. Then, tetramethylbenzidine was reacted for color development. Absorbance was measured at 370 nm in the microplate reader.

### 4.4. Flow Cytometry for Apoptosis Detection

With exposure to H_2_O_2_ for 24 h, apoptotic and necrotic appearances were evaluated using a flow cytometry with annexin V-FITC and propidium iodide (PI) staining (Annexin V-FITC Kit: Beckman Coulter, CA, USA). This staining is based on the principles that annexin V binds to phosphatidylserine expressed on the surface of apoptotic cells and that membrane-impermeable PI is incorporated into the DNA of necrotic cells. Both floating and attached cells were collected into a tube. The cell suspension were treated with 25 μL of annexin V-FITC and 12.5 μL of PI (6.25 μg/mL) for 10 min in the dark on ice. After being filtered with a cell strainer, the cell suspension was analyzed using a FACS Aria II system (Becton Dickenson, Franklin Lakes, NJ, USA). 

### 4.5. GSH Detection Assay

Cells were washed with PBS and incubated with 600 μL of 10 mM hydrochloric acid on each well. The cell lysate was obtained by repeating freeze–thaw cycles two times. The cell lysate was then mixed with 5% sulfosalicylic acid. After its centrifugation at 8000 g for 10 min, the supernatant was collected and dispensed in 40-μL batches into 96-well plates for the assay with a GSH detection kit (Total Glutathione Quantification Kit: Dojindo Molecular Technologies, Inc., Rockville, MD, USA). Briefly, after adding a buffer solution, the plate was incubated at 37 °C for 60 min. Then, a 5,5-dithiobis (2-nitrobenzoic acid) (DTNB) substrate and a GSH reductase were added to each well, and the plate was incubated at 37 °C for 10 min for chromogenic reaction of DTNB in the presence of GSH. The OD of DTNB in each well was measured using the microplate reader at 405 nm. Total GSH in each culture was calculated using a calibration curve for the standard GSH solution. To evaluate total GSH per unit cell in cell suspensions co-incubated with 5 mM NAC for 0, 3, 6, or 12 h in the 12 h preincubation, the values of total GSH were divided by cell numbers in the corresponding duplicated cell suspensions. To evaluate the total GSH in the cell culture after exposure to H_2_O_2_, the floating cells were also collected and combined with the adherent cells into a cells lysate.

### 4.6. Intracellular ROS Level

On day 2 after exposure to H_2_O_2_, cells were washed with PBS and incubated in 1000 μL of PBS containing 10 μM H_2_DCFDA (Thermo Fisher Scientific) for 15 min at 37 °C in a 5% CO_2_ atmosphere. H_2_DCFDA is membrane-permeable and intracellularly changes into fluorescent dichlorofluorescin diacetate (DCFDA) as a result of oxidization by ROS. The fluorescence intensity of DCFDA was measured using a multimode microplate reader (excitation: 490 nm; emission: 510–570 nm). The intracellular ROS level was determined according to the fluorescence intensity values, which were normalized against the amount of cells in a duplicate culture measured with a hematocytometer.

### 4.7. Gene Expression Analysis

Expressions for bone matrix-related genes were analyzed using RT-PCR on day 10. Total RNA in the cultures was extracted using the TRIzol reagent (Thermo Fisher Scientific) and a purification column (RNeasy, Qiagen, CA, USA). Following DNAse I (Ambion™ DNase I, Thermo Fisher Scientific) treatment, reverse transcription of 0.5 μg total RNA was performed using MMLV reverse transcriptase (Clontech, CA, USA) in the presence of oligo(dT) primer (Clontech). PCR on first-strand DNA was performed using Taq DNA polymerase (TaKaRa Ex Taq, Takara Bio, Shiga, Japan) to detect *col1a1*, *opn*, and *ocn* mRNA. Glyceraldehyde-3-phosphate dehydrogenase (*gapdh*) was employed as a house-keeping gene. The forward and backward primers (Sigma-Aldrich) were *col1a1*: 5′-GGCAACAGTCGATTCACC-3′ and 5′-AGGGCCAATGTCCATTCC-3′, *opn*: 5′-GATTATAGTGACACAGAC-3′ and 5′-AGCAGGAATACTAACTGC-3′, *ocn*: 5′-GTCCCACACAGCAACTCG-3′ and 5′-CCAAAGCTGAAGCTGCCG-3′, and *gapdh*: 5′-TGAAGGTCGGTGTCAACGGATTTGGC-3′ and 5′-CATGTAGGCCATGAGGTCCACCAC-3′. The PCR products were visualized on a 1.5% agarose gel by ethidium bromide staining under ultraviolet (UV) light.

### 4.8. Alkaline Phosphatase Activity

Alkaline phosphatase (ALP) activity of the culture was evaluated with azo dye staining. On day 3, cells were washed twice with Hanks’ solution and incubated with 120 mM Tris buffer (pH 8.4) containing 0.9 mM naphthol AS-MX phosphate and 1.8 mM fast red TR for 30 min at 37 °C. The ALP-positive area (%) on the stained images was calculated as (stained area/total dish area) × 100 using Image J.

### 4.9. Matrix Mineralization Assay

Matrix mineralization of the culture on days 14 and 21 was examined by quantification of total calcium deposition and Von Kossa staining to visualize the mineralized area in the culture, respectively.

For quantification of total calcium deposition, cells were washed with PBS and incubated overnight in 1 mL of 0.5 M HCl solution with gentle shaking. The solution containing the eluted calcium ions was mixed in an alkaline medium with *o*-cresolphthalein complexone (Calcium Assay Kit, Cayman Chemical Company, MI, USA), which reacts with calcium ions to form a purple cresolphthalein complexone complex. After gentle shaking, the OD for a cresolphthalein complexone was measured at 570 nm in the microplate reader. The amount of calcium deposition in each culture was calculated using a calibration curve for the standard calcium solution.

For Von Kossa staining, cells were fixed using a solution of 50% ethanol/18% formaldehyde solution for 30 min. The cultures were then incubated with 5% silver nitrate under UV light for 30 min. Finally, the cultures were washed twice with double-distilled H_2_O and incubated with 5% sodium thiosulfate solution for 2–5 min. The Von Kossa positive area (%), defined as (stained area/total dish area) × 100, was measured using an image analyzer.

### 4.10. Autologous Local Transplantation in A Massive Rat Femur Bone Defect

Autologous local transplantation in critical-size defects in rat femurs was performed based on the protocol in the previous study [34]. Eleven-week-old male SD rats were anesthetized with 2% isoflurane. A small unicortical hole penetrating to the bone marrow space was drilled 5 mm from the distal end of the epiphyseal region of the right femur. Approximately 500 μL of bone marrow was aspirated with a sterilized syringe and immediately suspended in the ODM. After collection of bone marrow, the muscle and the skin in the open wounds were sutured with 4-0 vicryl and 3-0 silk, respectively.

The harvested bone marrow tissue was plated on a 10-cm cell culture dish and incubated at 37 °C in a 5% CO_2_ atmosphere. After 24 h, the nonadherent cells were removed by washing with PBS. The culture medium was renewed at intervals of 3 days. After 14 days (when the cells reached 80% confluence), the cells were labeled with 1 nM fluorescent organic dot cell tracer (Long Term Cell Tracer 500 Cat. # P710G; 101 Bio.com, CA, USA) for 12 h in suspension. This cell tracer is known to be non-cytotoxic and is transmitted to daughter cells. Subsequently, cells were treated with 5 mM NAC for 3 h and then suspended again in a NAC-free GM. A bovine tendon-derived type I collagen sponge (Collaplug, Integra LifeSciences Corp, NJ, USA) [50] was used as a vehicle. The cell suspension was added into the inside of each sponge and incubated overnight at 37°C in a 5% CO_2_ atmosphere.

The left femur of the rat was subjected to a second auto-transplantation surgery. The rats were 13 weeks old at the time of the secondary surgery. After exposure of the left femur bone, a large rectangular segmental resection (5 × 5 × 2.5 mm), which was larger than the critical-sized defect in the rat femur previously reported [32,51], was made under irrigation in the center part of the cortical bone of the femur. The distal end of the defect was positioned 10 mm from the distal end of the epiphyseal region of the femur bone. Collagen sponges containing autologous osteoblast-like cells with and without NAC treatment were implanted into the segmental defect. The implantation site was wrapped with an absorbable collagen membrane (Koken Tissue Guide; Olympus Terumo Biomaterial, Tokyo, Japan). The femur bone was prevented from a fracture using a titanium splint and a glass fiber-containing hydraulic polyurethane resin-based dressing material. The muscle and the skin were sutured with 4-0 vicryl and 3-0 silk, respectively.

### 4.11. Micro-Computed Tomography Analysis

Three weeks after surgery, the left femur was excised and fixed in 10% neutral buffered formalin (Wako Pure Chemical) at 4 °C for 1 week. All splinting materials were removed during the fixation. Bone volume and mineral density of the regenerated bone at the site of the autologous cell implantation were evaluated using a ScanXmate-E090 device for three-dimensional micro X-ray computed tomography (micro-CT) (μCT 40, Scanco Medical AG, Bassersdorf, Switzerland) with an isotropic resolution of 8 μm. The femur specimens were X-rayed at an energy level of 70 kVp and a current of 114 μA. Grayscale images were processed using a Gaussian low-pass noise filter and threshold algorithms to distinguish between mineralized bone and the background. The area of interest for analysis was set on the combined area of the cortical and bone marrow space regions, which was defined as the region surrounded by connecting external basic lamellar points of both sides of the defect edges and the corresponding points on the opposite internal basic lamellar surface. The specific thresholds for bone tissue were determined by superimposing segmented images over the original grayscale images. A quantitative assessment was made of the following three dimensional parameters within the area of interest among groups: BV/TV (%), Tb. N (1/mm), Tb. Th (μm), and Tb. Sp (μm).

### 4.12. Histological Analysis

After micro-CT scanning, the samples were delipidated in ethanol and acetone and decalcified in a 10% EDTA disodium salt (EDTA 2Na) at 4 °C for 3 weeks. After dehydration in ethanol series, acetone, and xylene, samples were embedded in paraffin. Paraffin sections (3–5 μm) were stained with hematoxylin and eosin (H&E) or TRAP staining for morphological observation to check bone tissue structure and localization of the transplanted cells. For TRAP staining, the deparaffined sections were treated with a mixture of a tartaric acid solution and acid phosphatase substrates for 60 min at room temperature. Hematoxylin was used for counterstaining. Tissue morphology was observed in H&E and TRAP-stained sections under the bright-field setting on an all-in-one fluorescent microscope (BZ-9000, Keyence). The distribution of labeled transplanted cells was observed in the corresponding region of H&E-stained sections under the fluorescence setting with a 4′,6-diamidino-2-phenylindole filter (excitation: 400 nm; emission: 510 nm).

### 4.13. Statistical Analysis

One-way analysis of variance was used to assess the differences among multiple experimental groups. When appropriate, Dunnett’s test and Tukey’s HSD test were used as a post-hoc test. Student’s or Welch’s t-test was used to compare the two groups, and *p* < 0.05 was considered statistically significant. A statistical analysis was performed using IBM SPSS Statistics 21 statistical software (IBM Japan, Ltd., Tokyo, Japan).

## 5. Conclusions

Preconditioning of rat bone marrow-derived osteoblast-like cells with NAC enhanced the bone regeneration in autologous local transplants in femur critical-size cortical bone defects. The effects were associated with the prevention of oxidative stress-induced apoptosis and the restoration of osteogenic differentiation by reinforcing a cellular major antioxidant molecule, GSH. Transplanted osteoblast-like cells pretreated with NAC formed bone tissue directly and were finally embedded in osteocytic lacunae.
